# Case Report: A Novel Pathogenic Missense Mutation in *FAS*: A Multi-Generational Case Series of Autoimmune Lymphoproliferative Syndrome

**DOI:** 10.3389/fped.2021.624116

**Published:** 2021-03-18

**Authors:** Claudia L. Gaefke, Jonathan Metts, Donya Imanirad, Daime Nieves, Paola Terranova, Gianluca Dell'Orso, Eleonora Gambineri, Maurizio Miano, Richard F. Lockey, Jolan Eszter Walter, Emma Westermann-Clark

**Affiliations:** ^1^Department of Medicine, University of South Florida, Tampa, FL, United States; ^2^Cancer and Blood Disorders Institute, Johns Hopkins All Children's Hospital, Saint Petersburg, FL, United States; ^3^Department of Pediatrics, University of South Florida, Saint Petersburg, FL, United States; ^4^Hematology Unit, IRCCS Istituto Giannina Gaslini, Genoa, Italy; ^5^NEUROFARBA, Meyer Children's Hospital, Florence, Italy; ^6^Massachusetts General Hospital and Harvard Medical School, Boston, MA, United States

**Keywords:** ALPS (autoimmune lymphoproliferative syndrome), novel mutation, *Fas*, cytopenia, lymphoproliferation

## Abstract

Autoimmune Lymphoproliferative Syndrome (ALPS), commonly caused by mutations in the *FAS* gene, is a disease with variable penetrance. Subjects may be asymptomatic, or they may present with lymphadenopathy, splenomegaly, cytopenias, or malignancy. Prompt recognition of ALPS is needed for optimal management. We describe a multi-generational cohort presenting with clinical manifestations of ALPS, and a previously unreported heterozygous missense variant of uncertain significance in *FAS* (c.758G >T, p.G253V), located in exon 9. Knowledge of the underlying genetic defect permitted prompt targeted therapy to treat acute episodes of cytopenia. This cohort underscores the importance of genetic testing in subjects with clinical features of ALPS and should facilitate the reclassification of this variant as pathogenic.

## Introduction

Autoimmune lymphoproliferative syndrome (ALPS) is characterized by lymphadenopathy, splenomegaly, autoimmunity, especially autoimmune cytopenias, and an increased risk of lymphoma. Lymphoproliferation and autoimmunity result from failure of effector T-cells to undergo programmed cell death. The most common mutation associated with ALPS lies in the *FAS* gene, and is labeled as *ALPS-FAS*; other known mutations that cause ALPS include Fas ligand (*FAS-L)* and caspase 10 *(CASP 10)* (1, 2). Germline mutations in *FAS*, inherited in an autosomal dominant manner, comprise the largest group of ALPS cases, while somatic mutations in *FAS* are the second most common genetic cause of ALPS (2, 3). There is an expanding spectrum of ALPS-like disorders, including caspase 8 deficiency state (CEDS, resulting from a germline mutation in caspase 8 [*CASP8*]); RAS-associated autoimmune leukoproliferative disease (RALD), resulting from gain of function somatic mutation of the neuroblastoma RAS viral oncogene (*NRAS)* or proto-oncogene Kirsten Rat Sarcoma virus (*KRAS)*; X-linked lymphoproliferative syndrome (XLP1) resulting from mutation or deletion of the *SH2D1A* gene; heterozygous loss of function mutation in nuclear factor kappa light chain enhancer of activated B cells (*NFKB-1*), heterozygous loss of function mutation of cytotoxic T-lymphocyte associated protein 4 (*CTLA-4*), and mutations of the *FAS*-associated protein with death domain (FADD), among others. Approximately one-third of subjects with clinical features of ALPS have unidentified genetic defects (1, 2, 4). Patients with unknown genetic defects but clinical features of ALPS are classified as Dianzani autoimmune lymphoproliferative disease (DALD) (2).

Children with confirmed genetic findings and clinical features of ALPS require monitoring for cytopenias, splenomegaly, and malignancy as they age. A sizeable proportion of subjects with ALPS-FAS (74%) require medical/surgical intervention during their lifetime, and intervention is often initiated due to autoimmune cytopenias, massive splenomegaly, or adenopathy (3).

The variable clinical phenotype of ALPS contributes to the complexity of management. Affected subjects can carry a pathogenic variant but be relatively asymptomatic, and biomarkers for development of lymphoproliferation and autoimmunity have not been fully established. It is challenging to determine when to initiate immunomodulating therapy with milder disease, such as intermittent thrombocytopenia. Targeted therapy, including T-cell modulation with sirolimus, can be beneficial, but timing and duration of therapy is unclear (5, 6). Splenectomy should be avoided because of the high risk of subsequent sepsis, increased risk of death, and failure to prevent recurrence of autoimmune cytopenia in many subjects (3, 7). Here we describe a family with a novel *FAS* mutation found across several generations and discuss challenges in long-term management.

## Definition of ALPS

For this study, ALPS was defined as proposed by Oliveira et al. (2). These diagnostic criteria divide symptoms and laboratory values into required and accessory criteria. Required criteria include chronic (>6 months), non-malignant, non-infectious lymphadenopathy or splenomegaly or both, and elevated CD3+ T-cell receptor (TCR)αβCD4-CD8- double negative cells (DNTCs) (≥1.5% of total lymphocytes or 2.5% of CD3+ lymphocytes) in the setting of normal or elevated lymphocyte counts. Accessory criteria are divided into primary accessory [(1) defective lymphocyte apoptosis in two separate assays; (2) somatic or germline pathogenic mutation in *FAS, FASLG*, or *CASP10*] and secondary accessory criteria which include useful biomarkers (elevated soluble FAS-ligand [sFASL], interleukin-10 (IL-10), vitamin B12, interleukin-18 (IL-18) levels, elevated immunoglobulin G (IgG) levels), typical immuno-histological findings, autoimmune cytopenias (which must be accompanied by elevated IgG), or family history of non-malignant/non-infectious lymphoproliferation with or without autoimmunity. A definitive diagnosis is based on the presence of both required criteria plus one primary accessory criterion; a probable diagnosis is based on the presence of both required criteria plus one secondary accessory criterion (2).

## Case Presentation

**Figure 1:**
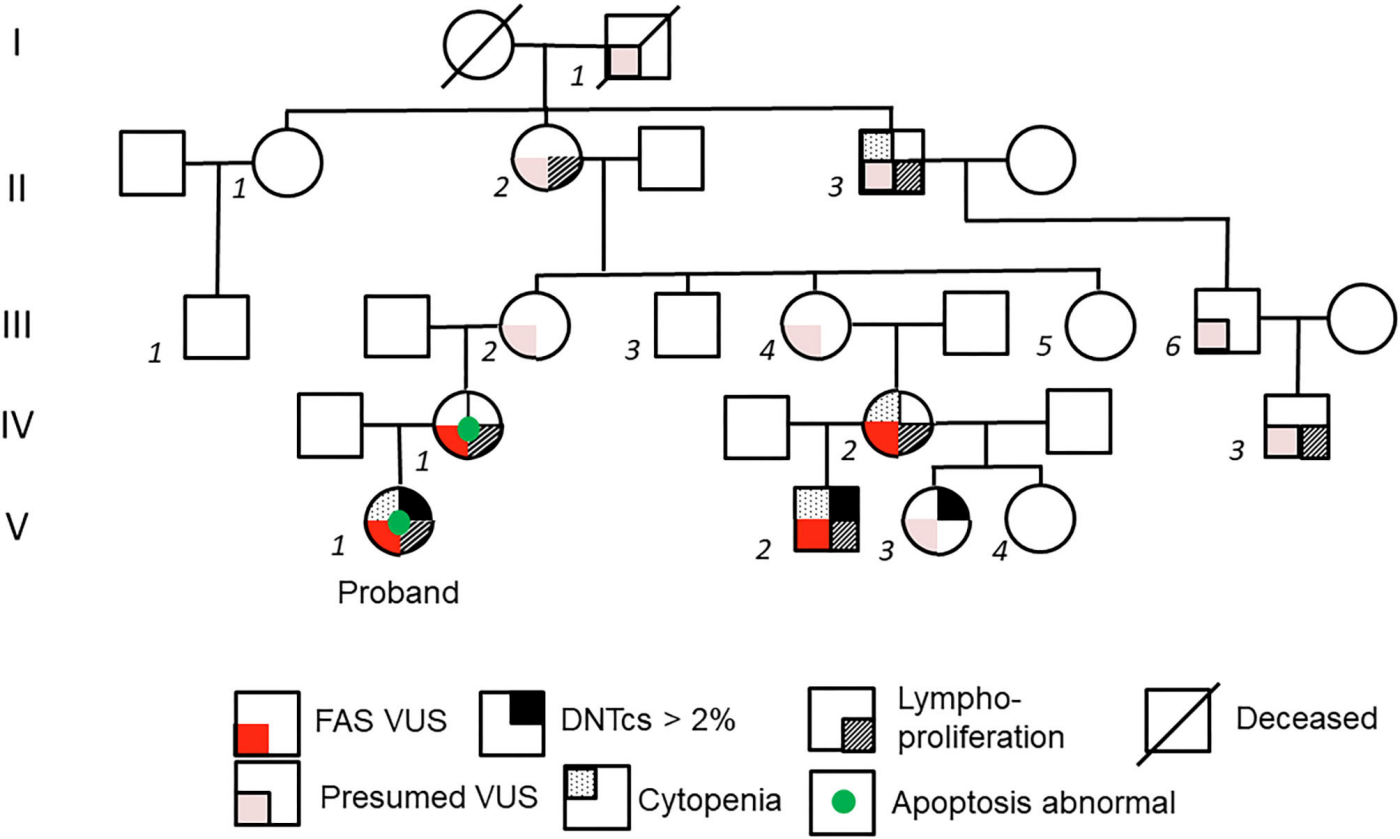
Pedigree of kindred. On left axis, generations are labeled with Roman numerals I-V. Individual members of the family are labeled with italicized Arabic numerals. Family members related only by marriage are not numbered. Detailed family history was obtained from subject II-2, the maternal great-grandmother of the proband V-1. Subject II-2 postulates that the *FAS* variant stems from her father's side of the family (I-1) because his sister (not depicted) died of leukemia at age 17.

The proband (V-1) (Figure 1) is a 7-month-old female who presented to clinic with greater than 6 months of mild anterior and posterior cervical and axillary lymphadenopathy, with the largest lymph node measuring 1 cm in diameter in the neck. She also had a spleen of 7.5 cm in length, slightly larger than normal for age, with the upper limit being 7 cm in length for a 12-month old (8). She had a history of asthma triggered by viral respiratory tract infections and no history of recurrent or severe bacterial sinopulmonary infections. She was normal developmentally for her age. Complete blood count (CBC) with differential was normal at presentation, but at 2 years of age she experienced an episode of autoimmune hemolytic anemia, with hemoglobin nadir of 5.3 g/dL and mild thrombocytopenia (platelets 108,000). Immune evaluation was notable for normal immunoglobulin levels and normal diphtheria and tetanus antibody titers; however, pneumococcal titers were only protective for 50% of a 10-serotype panel, and patient had a low *Haemophilus influenzae* type B (Hib) titer (Table 1).

**Table 1:** Immunophenotypic evaluation of pediatric subjects.

		**Subject**			**Subject**	
**Evaluation (units)**	**Normal values for age 12 mo**	**V-1**	**V-2**	**Normal values for age 6-8 years**	**V-3**	**V-4**
Age at evaluation		13 months	12 months		6 years	8 years
IgG [mg/dL]	246–904	895				
IgA [mg/dL]	27–66	73				
IgM [mg/dL]	40–143	56				
IgE [IU/mL]	0–200	5.2				
Pneumococcal Titers	>50% protective	5/10				
Diphtheria [IU/mL]	≥0.01	1.83				
Tetanus [IU/mL]	≥0.01	2.83				
Hib [mg/dL]	1	0.81				
WBC [10*3/mm^3^]	6.4–13.0	9.9	6.14	4.4–9.58	6.73	6.2
Hgb [g/dL]	10–13.2	11.2	10.1	10.9–14.9	13.5	14.8
MCV [fL]	70–90	80	30.3	76–98	78	85
PLT [10*3/mm^3^]	150–450	214	129	150–450	250	264
ANC [cells/mm^3^]	500–9500	2400	1140	1500–7500	3810	3420
Abs Lymph [cells/mm^3^]	3400–9000	7131	6387	1900–3700	2513	2300
Abs CD3 [cells/mm^3^]	1900–5900	5027	4747	1200–2600	2042	1754
CD4:CD8 [cells/mm^3^]	1.34–3.04	2.20	1.23	1.18–2.65	0.97	1.2
Abs CD4 [cells/mm^3^]	1400–4300	3170	2417	650–1500	844	877
Abs CD8 [cells/mm^3^]	500–1700	1397	1956	370–1100	867	738
Abs CD56 [cells/mm^3^]	160–950	267	243	110–480	205	197
Abs CD19 [cells/mm^3^]	610–2600	1745	1369	270–860	261	441

*Ig, immunoglobulin; Hib, Haemophilus influenzae type b; WBC, white blood cell; Hgb, hemoglobin; MCV, mean cell volume; PLT, platelets; ANC, absolute neutrophil count; Abs, absolute; Lymph, lymphocytes. Age-appropriate ranges were identified for immunoglobulins (9), CBC (10), lymphocyte subsets (11) and CD4:CD8 ratio (12)*.

Her mother (IV-1 in Figure 1) reported a personal history of splenomegaly, which had been diagnosed along with “kidney enlargement” during her pregnancy, and a family history of splenomegaly, lymphadenopathy, and hematologic malignancy in prior generation maternal relatives. The grandmother of subject IV-1, subject II-2, reported a personal history of neck lymphadenopathy requiring excision during her late teenage years. Subject II-2 suspected that she had inherited her tendency toward lymphadenopathy from her father (I-1 in Figure 1), whose sister had died of leukemia at age 17.

Among living family members with suspected ALPS, a 17-month-old male second cousin (V-2) was also seen in the clinic for persistent lymphadenopathy. Subject V-2 had posterior cervical lymphadenopathy, splenomegaly, and history of thrombocytopenia. There was no history of recurrent or severe infections, bleeding, or other illnesses. His mother (IV-2) reported a personal history of recurrent lymphadenopathy, splenomegaly since childhood, and chronic anemia with no formal history or testing for ALPS. Subject IV-1 and IV-2 (mothers to proband and V-2, respectively) share a maternal grandmother (II-2), who reported neck lymphadenopathy in her teenage years, as mentioned previously. This maternal grandmother (II-2) had a sister (II-1) who was healthy, and a brother (II-3) who required splenectomy at age 15 and often had abnormal blood cell counts in childhood. A child of II-1, (male child III-1), was healthy. II-3 had a son, III-6, who was healthy, but his grandson IV-3 required splenectomy at 1 year of age due to splenomegaly. Subject II-2 also had four children (subjects III-2, III-3, III-4, and III-5) who did not show notable signs of ALPS. Based upon this information, further evaluation was pursued in available family members, which included two half-siblings of subject V-2: subjects V-3 and V-4. Subject V-3, a 6-year-old female, had a history of recurrent oral lesions resembling herpes simplex virus with no clinical evidence of ALPS. Subject, V-4, an 8-year-old female, had a history of recurrent ear infections and otherwise no clinical history suggestive of ALPS.

## Laboratory Evaluation and Results

Given this clinical multi-generational history, laboratory evaluation of the kindred was undertaken to identify a possible shared mutation leading to ALPS.

The proband and her mother were evaluated for ALPS. As noted above, the proband had a relatively normal immune phenotype except for low recall response to certain vaccine antigens. Proband's mother (IV-1) had mild leukocytosis on CBC but normal absolute lymphocytes (3100 cells/μL), and full immune phenotyping was not performed. Both proband and mother had an abnormal apoptosis assay; proband (V-1) had FAS activity of 38% of control, and mother (IV-1) had FAS activity 41% of control (normal 68–93%). Flow cytometry in V-1 showed >2% TCR αβDNTcs (Cincinnati's Children's Hospital ALPS Flow Cytometry panel, Cincinnati, OH) which prompted further evaluation for accessory criteria. Secondary accessory criteria, including clinical and serological markers, were met as noted on Table 2. For confirmation, genetic sequencing was pursued using a 207-gene Primary Immunodeficiency Panel (Invitae, San Francisco, CA). Genetic sequencing confirmed a mutation in *FAS*, c.758G>T (p.G253V), exon 9, in the death domain. This mutation was identified in both the proband and her mother. This sequence change replaces glycine with valine at codon 253 of the FAS protein (p.Gly253Val). The glycine residue is moderately conserved and there is a moderate physicochemical difference between glycine and valine. This variant is not reported in the literature with FAS-related conditions and is not present in Genome Aggregation Databases (gnomAD June 2020, no allele frequency). The Genome Aggregation Database (gnomAD), also known as ExAC in its first release, does not include individuals with G253V amino acid substitution mutations. It does include 5 individuals with a nearby p.His285Arg mutation among 251,308 alleles sequenced.

**Table 2:** Family members evaluated based on ALPS Criteria.

	**Subject**					
**ALPS Criteria**	**V-1**	**V-2**	**V-3**	**V-4**	**IV-1**	**IV-2**
**Required:**						
TCR αβDNTcs >2% or >68 cells/μL	Present	Present	Present	Absent	N/A	N/A
Clinical: LAD and/or Organomegaly	Present	Present	Absent	Absent	Present	Present
**Primary accessory:**						
Genetic sequencing	HMM	HMM	Declined Testing	Declined Testing	HMM	HMM
	FAS c.758 G>T (p. Gly253 Val)	FAS c. 758 G>T (p. Gly253 Val)	N/A	N/A	FAS c. 758 G>T (p. Gly253 Val)	FAS c. 758 G>T (p. Gly253 Val)
Apoptosis assay (normal 68-93%)	38%	N/A	N/A	N/A	41%	N/A
**Secondary accessory:**						
IL-18 (normal <540 pg/mL)	1513	958	714	260	N/A	N/A
IL-10 (normal <2 pg/mL)	300	186	40.5	<1.6	N/A	N/A
sFAS-L (normal 69-492 pg/mL)	5952	4825	1601	169	N/A	N/A
Vitamin B12 (normal 200-1100 ng/mL)	>2000	>2000	1892	675	N/A	N/A
Clinical manifestations	AIHA	ITP	Absent	Absent	History of anemia	History of Anemia
Family history	LP, AI	LP, AI	LP, AI	LP,AI	LP, AI	LP, AI

*AI, autoimmunity; AIHA, autoimmune hemolytic anemia; DNTCs, double negative T cells; HMM, heterozygous missense mutation; IL, interleukin; ITP, immune thrombocytopenia; LAD, lymphadenopathy; LP, lymphoproliferation; N/A, not applicable; sFAS-L, soluble FAS ligand*.

Laboratory evaluation for subjects V-2, V-3, and V-4 (second cousins of the proband) and their mother (IV-2) was performed. Subjects V-2, V-3, and V-4 did not undergo full immune phenotyping, but CBC and lymphocyte subsets, along with age-appropriate normal ranges, are reported in Table 1. ALPS panel testing was performed in V-2, V-3, and V-4. V-2 and V-3 had >2% TCR αβDNTcs and serological markers consistent with ALPS, while subject V-4 did not meet criteria for ALPS (Table 2). Subject IV-2 declined genetic sequencing for subject V-3 and V-4, but genetic sequencing in subject V-2 confirmed the same variant. Both mothers (subjects IV-1 and IV-2) did not wish to undergo immune phenotyping, serologic markers, or ALPS panel, but chose to pursue genetic testing; the same genetic variant was confirmed.

## Evolution of Clinical Phenotype and Treatment

**Figure 2:**
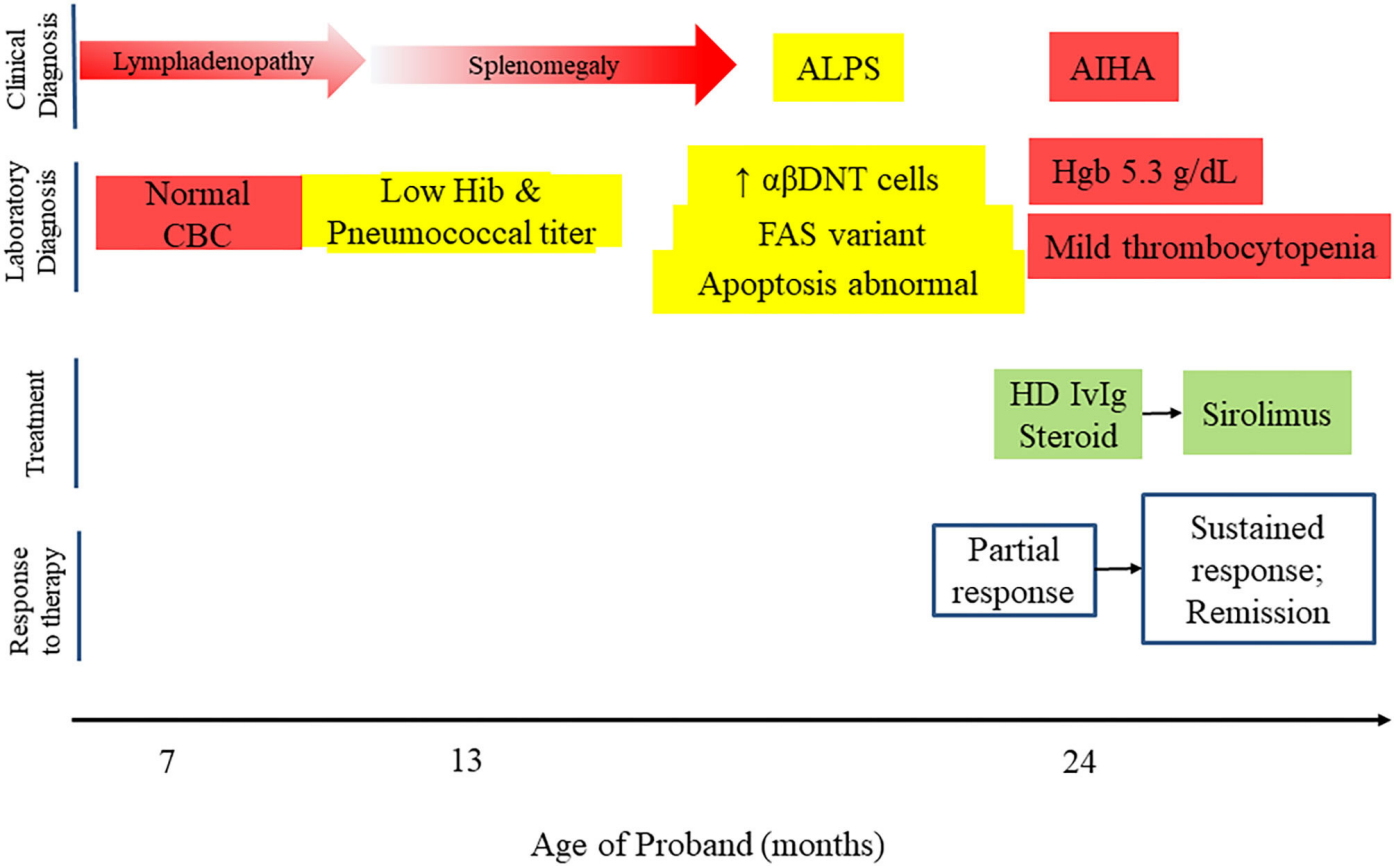
Timeline of proband's clinical course, including clinical and laboratory diagnosis, treatment and response to therapy.

One year after initial presentation, the proband developed an acute episode of autoimmune hemolytic anemia, initially treated with corticosteroids and high dose intravenous immunoglobulin (IVIG). Since she had an established diagnosis of ALPS, targeted therapy with sirolimus was initiated at 2 mg/m^2^. She was able to discontinue corticosteroids and has not required further high dose IVIG or hospitalization (Figure 2). The family elected to discontinue sirolimus after 1 year of treatment, and she is currently being monitored for recurrence of cytopenias or progression of ALPS.

Subject V-2, who met criteria for ALPS, was also found to have intermittent thrombocytopenia, with platelet counts between 84,000 and 200,000/μL. He is being monitored with plans to initiate sirolimus for platelet counts <50,000/μL or other persistent cytopenias.

Subject V-3 is being monitored for clinical manifestations of ALPS, as she meets required and secondary accessory criteria, but parents have declined genetic sequencing.

Subject V-4 did not manifest clinical or laboratory features of ALPS, and parents declined genetic sequencing.

Subjects IV-1 and IV-2, both of whom carry the VUS in *FAS*, are being monitored for further clinical manifestations of ALPS.

## Discussion

Autoimmune lymphoproliferative syndrome is a disease with variable penetrance from asymptomatic to lymphadenopathy, splenomegaly, cytopenias, and malignancy. Though most patients develop lymphoproliferation at a median age of 11.5 months, seemingly unaffected family members with heterozygous *FAS* pathogenic variants are also predisposed to malignancy, most commonly non-Hodgkin lymphoma later in life, with a lifetime risk of up to 20% (13, 14). This underscores the importance of recognizing ALPS promptly to appropriately manage complications and to monitor asymptomatic kindred for prompt recognition of malignancy which may present later in life.

When the proband met criteria for ALPS, it was decided to pursue screening for willing kindred. By doing this, a second family member (subject V-2) was found to meet criteria for ALPS. A third family member (V-3) has not developed the required clinical manifestations of ALPS but does meet many laboratory benchmarks for ALPS diagnosis, including elevated DNTCs, IL-18, IL-10, sFAS-L, and vitamin B12. In total, four of the six family members agreed to genetic testing, and all four were found to share the same VUS in *FAS*.

The missense mutation identified by the genetic testing company (Invitae) that performed the 207-gene primary immunodeficiency panel was labeled as a “variant of uncertain significance (VUS)” because symptomatic individuals with this specific variant have not previously been reported in the literature or in population databases. The absence of the variant in population sequence databases increases the likelihood that it is pathogenic. A stop codon in the same residue G253V has been previously reported, with a FAS-induced apoptosis of 25% of control, slightly less than the apoptosis activity reported in this study (38–41%) (15). There have been multiple *FAS* mutations reported in the death domain of exon 9, and these mutations can show clinical variability among family members, including asymptomatic members with the mutation and diminution of signs and symptoms with increasing age (15, 16). The cohort described in this paper shows a similar pattern of clinical presentation, with the most affected subjects presenting during childhood. Taken together, the novel missense mutation, apoptosis assay/ALPS panel, and absence of the variant in population databases provide moderate to strong evidence that this variant should be reclassified as pathogenic, according to published guidelines for the interpretation of sequence variants (17). The clinical phenotype and cosegregation of the gene in multiple affected family members provide additional supporting evidence (17).

Confirmation of ALPS with genetic sequencing facilitates clinical use of targeted therapies for complications of ALPS. Several potential therapies exist, including corticosteroids, IVIG, rituximab, mycophenolate mofetil (MMF), and mammalian target of rapamycin (mTOR) inhibitor therapy such as sirolimus (18). While the proband's autoimmune hemolytic anemia was initially treated with corticosteroids and intravenous immunoglobulin, due to the genetic diagnosis, she was successfully transitioned to sirolimus, minimizing steroid exposure. This is not surprising given published reports of durable complete response of autoimmunity, lymphadenopathy, and splenomegaly within 3 months of sirolimus initiation in ALPS subjects, as well as no detection of DNTCs in most subjects (19). Though rituximab is often used as a second-or third line option for pediatric subjects with autoimmune cytopenia, the risk of B-cell depletion and prolonged hypogammaglobulinemia is a concern, and long-term management with corticosteroids is not ideal. Splenectomy for autoimmune cytopenia is a last resort, given the risk of sepsis and failure to prevent recurrence of cytopenias in many subjects post-splenectomy (7). Mycophenolate mofetil (MMF) is generally well-tolerated and inactivates a key enzyme in purine synthesis required for lymphocyte proliferation (20, 21). However, MMF does not reduce DNTCs, which may account for suboptimal results such as a partial response or relapse in some ALPS subjects (13). Sirolimus shows good responses in ALPS subjects with autoimmune cytopenias, with possible side effects including hypercholesteremia, hypertension, and mucositis (22). Monitoring of clinical symptoms and ALPS biomarkers can be used to decide when and how treat patients with confirmed disease, as these biomarkers can increase as disease becomes active. DNTCs tend to decrease when patients are treated with sirolimus, and monitoring of DNTC percentage can therefore be useful. Additional serological biomarkers to monitor disease activity and treatment response include vitamin B12 levels, soluble FAS ligand, interleukin-10, and interleukin-18 levels. It is important to note that achieving the target plasma levels of sirolimus is not necessary to control the disease, as levels can be below 5 ng/mL and still be effective for disease control (23, 24). Additionally, in pediatric subjects who show decreased disease activity and eventually go into remission as they reach adolescence, sirolimus discontinuation can be considered, and the medication can be restarted if relapses occur.

We trust that this report will facilitate reclassification of this *FAS* variant as pathogenic and underscore the importance of genetic testing, which is useful for targeted therapy, family planning, and monitoring patients for malignancy.

## Data Availability Statement

The original contributions presented in the study are included in the article/supplementary material, further inquiries can be directed to the corresponding author/s.

## Ethics Statement

The studies involving human participants were reviewed and approved by Johns Hopkins Institutional Review Board IRB00103900. Written informed consent to participate in this study was provided by the participants' legal guardian/next of kin. Written informed consent was obtained from the individual(s), and minor(s)' legal guardian/next of kin, for the publication of any potentially identifiable images or data included in this article.

## Author Contributions

CG, JM, and JEW conceived the presented idea. CG, EW-C, JEW, and JM verified the methods used and reviewed the clinical information presented. CG, JM, DI, EW-C, and JEW assisted with data collection and manuscript review and editing. EG provided guidance regarding long-term management of subjects and serologic monitoring. EW-C oversaw the writing, data collection, and editing process. RFL provided critical review of the manuscript. MM, PT, and GD performed apoptosis assay on a research basis. JEW encouraged to describe this cohort's findings and supervised the findings of this work. All authors discussed the results and contributed and agreed to the final manuscript.

## Conflict of Interest

The authors declare that the research was conducted in the absence of any commercial or financial relationships that could be construed as a potential conflict of interest.
